# Niche diversification of Mediterranean and southwestern Asian tortoises

**DOI:** 10.7717/peerj.13702

**Published:** 2022-07-11

**Authors:** Daniel Escoriza, Jihene Ben Hassine

**Affiliations:** 1 GRECO, University of Girona, Girona, Spain; 2 University of Tunis el Manar, Tunis, Tunisia

**Keywords:** Ecology, Evolution, Reptiles, Testudo, Glacial cycles, Refugia

## Abstract

**Background:**

Tortoises of the genus *Testudo* are widely distributed throughout the Mediterranean region and southwestern Asia. However, the evolutionary mechanisms of diversification in this genus are still poorly understood.

**Methods:**

In this study, we assessed the evolutionary patterns in the climate niches of five species and 11 subspecies of the genus *Testudo* using ecological niche models and evaluated the niche overlap based on species phylogenetic distances.

**Results:**

The ecological models indicated that most species differ in their climate niches, but show overlap, with gradual transitions at range boundaries. As expected, the ecological divergence among subspecies was lower than that among species. Evaluation of the phylogenetic signal indicated that climate niches have been weakly conserved, but sister species also show high evolutionary divergence.

## Introduction

Closely related species tend to respond similarly to environmental variation because they share similar life histories and physiological traits (Cruz et al., 2011; Dahlke et al., 2020). This lineage-specific evolutionary inertia in environmental response is known as phylogenetic niche conservatism (Crisp & Cook, 2012). Niche conservatism is common during the speciation process of a lineage, and it is produced by the fragmentation of ancestral subpopulations in environmentally analogous regions (Peterson & Nyari, 2008). Climatic oscillations, including glacial phases, promote niche diversification, as some subpopulations are subject to substantial contractions in their ranges during unfavourable periods, reducing or preventing gene flow (Leroy et al., 2017; Andersen et al., 2019).

A vicariant mode of speciation is evident in several lineages of Mediterranean reptiles (Kindler et al., 2017; Senczuk et al., 2017). Most reptiles are ectothermic, having entire reliance on external heating sources to control body temperature and complete embryonic development (Brattstrom, 1965; Booth, 2006). Therefore, the temperature is a major environmental factor influencing species richness among reptile assemblages in temperate regions (Rodríguez, Belmontes & Hawkins, 2005). The oscillatory phases throughout the Pleistocene, which involved a general drop in temperature and increased aridification (Magri & Parra, 2002), had profound impacts on the process of reptile diversification and distribution (Araújo et al., 2008; Fitze et al., 2011; Anadón et al., 2015). However, the influence of recent climatic history on the diversification process of Mediterranean tortoises is little known.

In this study, we tested for phylogenetic climatic niche conservatism and modelled the ranges of the tortoise species of the genus *Testudo*. This genus includes species that are morphologically similar (size ranges between 20 and 35 cm) and are generalist herbivores (Bonin, Devaux & Dupré, 2006). The genus *Testudo* comprises five species and 15 subspecies, distributed throughout Eurasia and North Africa in open and relatively warm environments (Calzolai & Chelazzi, 1991; Fritz et al., 2009; Escoriza et al., 2022). Most *Testudo* species are either parapatrically or allopatrically distributed, although some species are sympatric in a partial range of their distribution in the southern Balkans, such as *T. graeca-T. hermanni* and *T. hermanni-T. marginata* (Valakos et al., 2008). This pattern of occurrence suggests: (i) that the climatic niche of the species is conserved, being isolated by competitive exclusion or environmental barriers (Pasch, Bolker & Phelps, 2013; Jankowski et al., 2013; Li, Shao & Li, 2020); or (ii) that the species are ecologically divergent but contact in narrow transitional areas within the margins of environmental tolerance of the two parapatric species (Jones et al., 2020). We tested these hypotheses using ecological niche models (ENMs).

## Materials & Methods

### Study region and species

**Figure 1:**
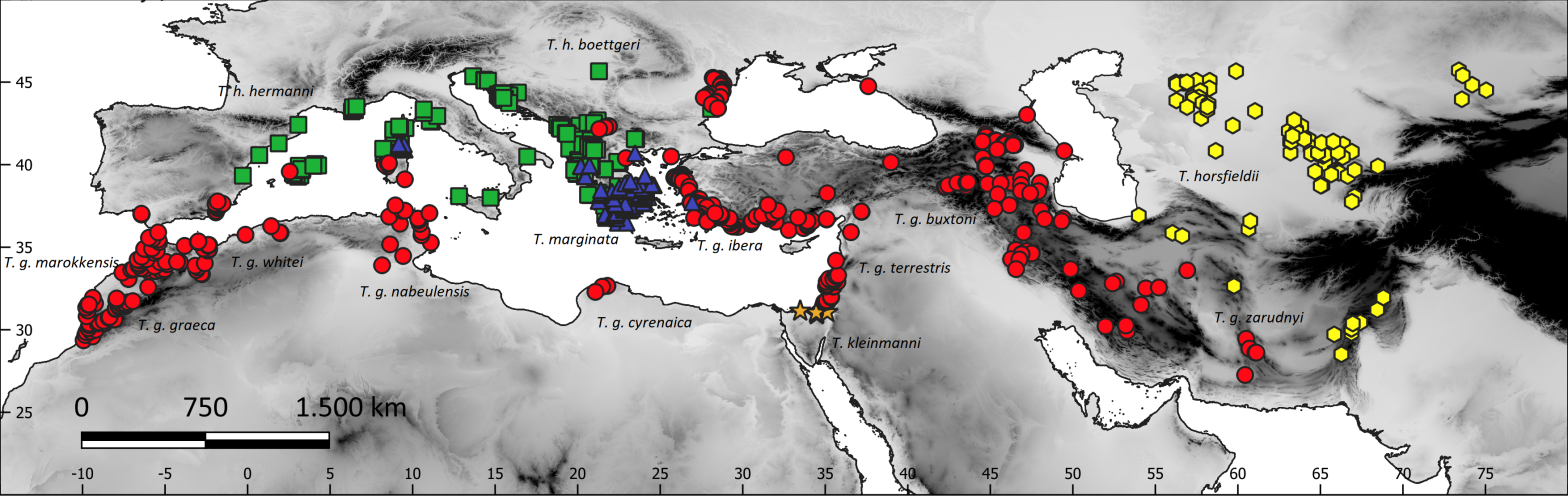
The study region and species sites, with superimposed terrain elevation (grayscale). Red circles, *Testudo graeca*; green squares, *Testudo hermanni*; yellow hexagons, *Testudo horsfieldii*; orange stars, *Testudo kleinmanni*; blue triangles, *Testudo marginata*.

The study area included the western Palaearctic biogeographic realm (southern Europe and northern Africa) and southwestern Asia between Pakistan and Turkey (Fig. 1). Data on the occurrence of all the five species of the genus *Testudo* were obtained during several samplings in the region (Escoriza & Ben Hassine, 2017; Escoriza & Pascual, 2021), from bibliographic sources, the GBIF (Global Biodiversity Information Facility, https://www.gbif.org) and iNaturalist (only *Testudo marginata*; https://www.inaturalist.org/) databases (Appendix S1 and S2). These databases are to be reliable data sources for biodiversity research (García-Roselló et al., 2015; Hochmair et al., 2020). We selected from the GBIF those records added since 2000, with a fine spatial resolution (error < 1,000 m), far from cities and within the known ranges of the species, as defined by Bonin, Devaux & Dupré (2006). To reduce spatial autocorrelation bias (Boria et al., 2014), record points that are closer than 10 km from each other were removed from the analysis using spThin (Aiello-Lammens et al., 2015) in R (R Core Team, 2022). This process reduced the number of locations by 54% (from 964 to 519) (Appendix S1): *T. graeca* (274 records), *T. hermanni* (103), *T. horsfieldii* (77), *T. kleinmanni* (4), and *T. marginata* (61) (Fig. 1).

**Figure 2:**
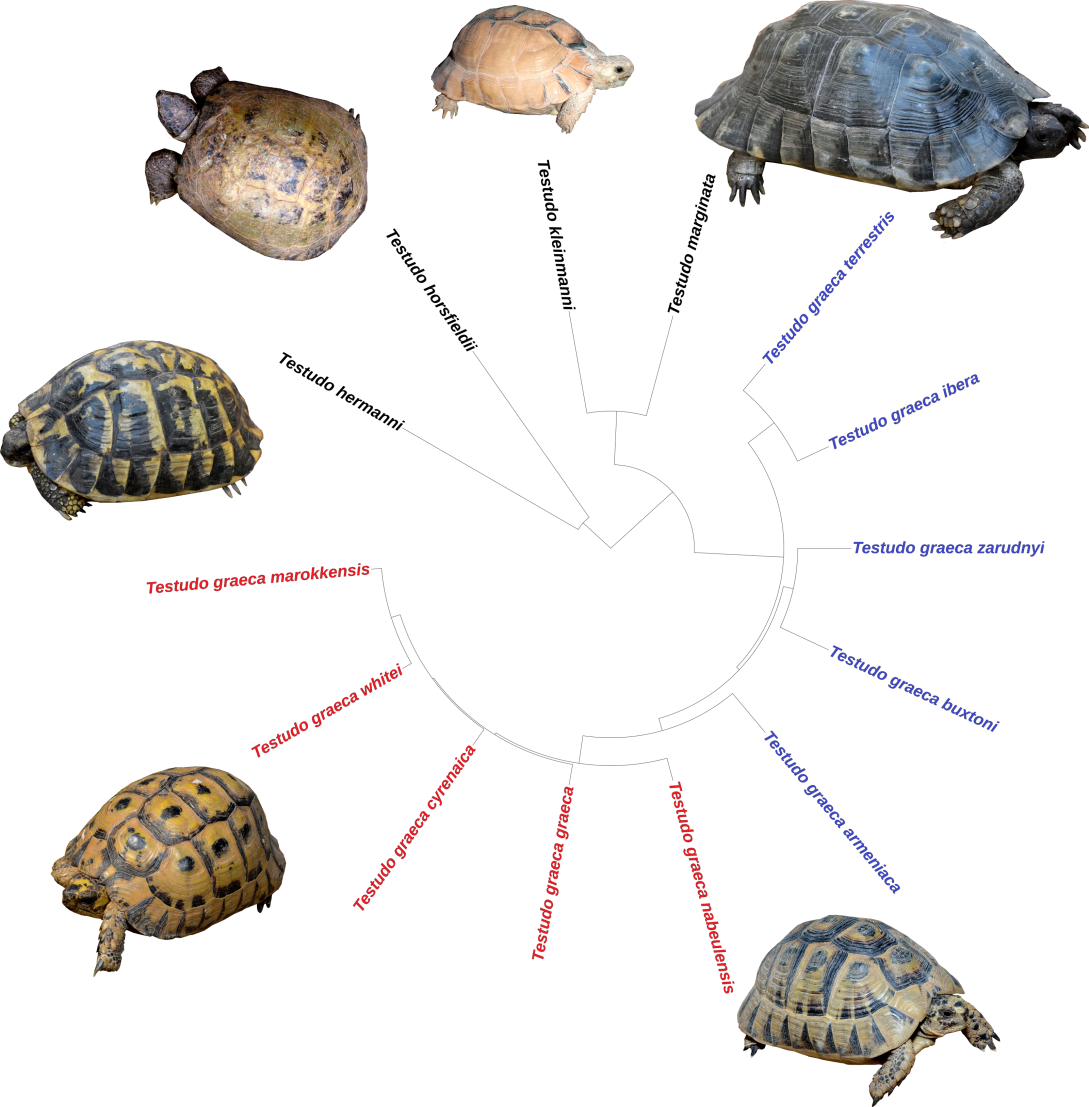
Phylogenetic relationships among species and subspecies of the genus *Testudo*, based on estimated times of molecular divergence. In *T. graeca* the names in red represent the western group of subspecies and in blue the eastern group. Clockwise: *Testudo marginata* (blackish specimen), *T. graeca nabeulensis*, *T. graeca whitei*, *T. hermanni hermanni*, *T. horsfieldii*, *T. kleinmanni*.

**Figure 3:**
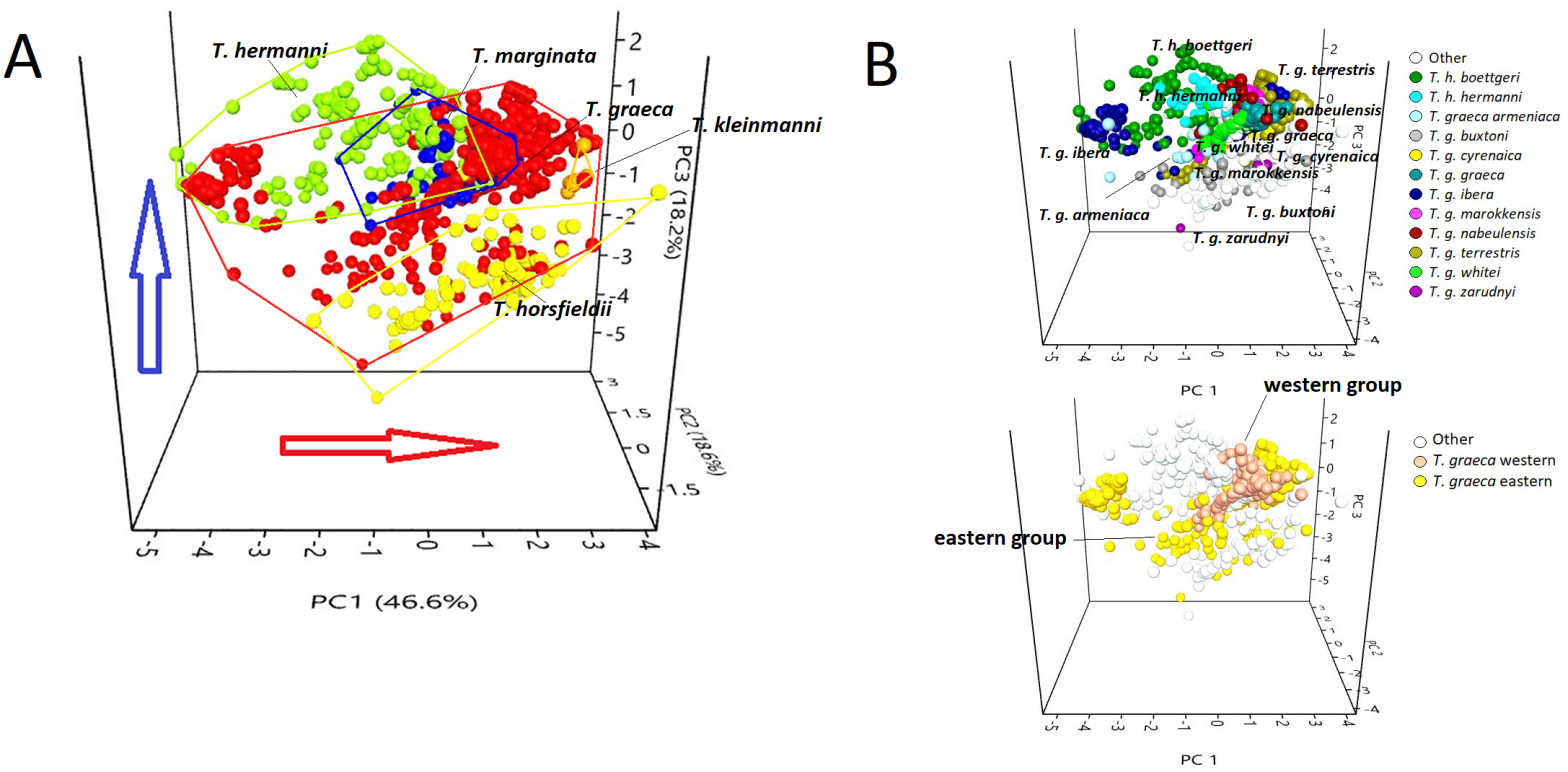
PCA triplot showing (A) the position of each species of the genus *Testudo* and (B) the position of the subspecies (above) and *T. graeca* western-eastern subclades (below), based on data obtained from bioclimatic variables. Red circles, *Testudo graeca*; green circles, *Testudo hermanni*; yellow circles, *Testudo horsfieldii*; orange circles, *Testudo kleinmanni*; blue circles, *Testudo marginata*. The red arrow indicates an increase in temperature; the blue arrow indicates an increase in rainfall.

The taxonomic classification of Rhodin et al. (2021) was used *i.e.,* only considering as valid those subspecies genetically and morphologically divergent. The niche analyses evaluated the ecological differentiation among species and subspecies, but also of two well-supported subclades within the genus (Fritz et al., 2007; Graciá et al., 2017): the eastern (Asian) group of subspecies of *T. graeca* (*T. g. armeniaca, T. g. buxtoni, T. g. ibera, T. g. terrestris, T. g. zarudnyi*) and the western (African) group (*T. g. cyrenaica, T. g. graeca, T. g. marokkensis, T. g. nabeulensis, T. g. whitei*) (Figs. 2 and 3).

### Environmental data

Data on 19 bioclimatic variables, available in World Clim2 (Fick & Hijmans, 2017), were obtained from the species occurrences. Model complexity was reduced by estimating the increase in the variance inflation factor (VIF) (Craney & Surles, 2002). To estimate the VIF, we defined buffer polygons of 200 km around the location of each species and generated 1000 random points within these polygons. The climate data obtained for the random points and the location of each species were included in a logistic regression and used to estimate the variations in VIF for each new variable added to the model. We started with a simple model including only bio 10 (mean temperature of the warmest quarter), bio 11 (mean temperature of the coldest quarter), and bio 12 (annual precipitation). These variables were initially chosen because of their relevance in the life cycles of tortoises, and they included summer and winter temperatures (specifically associated with embryonic development and hibernation) and moisture availability (Lambert, 1983; Anadón et al., 2012). Variables with VIF > 10 were excluded (Schroeder, Lander & Levine-Silverman, 1990).

### Data analyses

We visualized the realised niche of each species using principal component analysis (PCA) of the normalised variables. The climate niche of each species was generated using Maxent, as this method is suitable for models based only on presence data (Elith et al., 2011). The best Maxent model was selected by comparing several candidates built using various features (L: linear; Q: quadratic; H: hinge; P: product; T: threshold) and regularisation multipliers (RMs). This iterative selection was conducted using the Akaike criterion corrected for finite samples (AICc), using the ENMeval functions (Muscarella et al., 2014) in R. The features and the RM for the optimal model were used to generate 30 replicates in Maxent 3.4.4 (Phillips, Anderson & Schapire, 2006), calibrated using 75% of the species occurrences. The reliability of these models was assessed using the AUC statistic and variable contributions based on permutations of the presence–background data (Phillips, Anderson & Schapire, 2006).

The best ENMs were used to infer the location and extent of potential palaeoclimate refugia. To do this, we projected the current relationships between climate and species to the paleoclimatic conditions, using the default clamping function in Maxent (Zhao et al., 2021). We used several palaeoclimatic layers covering the period from the early Pleistocene (ca. 787 ka), and a complete glacial cycle that included the last interglacial (ca. 130 ka), the last glacial maximum (ca. 21 ka), and the mid-Holocene (ca. 6 ka) (Otto-Bliesner et al., 2006; Brown et al., 2018). The logistic result of these models was binarised using the fuzzify function in Quantum GIS (mid point = 0.5). The four layers generated (along with one generated for present conditions) were summed, producing a new layer in which values close to five identified those areas that were climatically suitable as refugia throughout most of the Pleistocene–Holocene periods. The niche of *T. kleinmanni* was not modelled, because the number of localities was low.

We also evaluated the niche divergence between pairs of species and subspecies of the genus *Testudo*. These comparisons were conducted for all species and between parapatric conspecific subspecies. Those taxa having fewer than 19 occurrences were excluded from the niche tests (Jenkins & Quintana-Ascencio, 2020). Niche divergence was estimated using a suite of tests (overlap, linear and blob range-breaking, and identity and background tests) that estimated the D index, which varies between 0 (no overlap) and 1 (full overlap) (Schoener, 1970). The overlap test quantified the similarity between ENMs using Latin hypercube sampling (Warren et al., 2021). The range-breaking tests assess the occurrence of sharp changes in environmental conditions at the range boundaries of a species. The linear test assumes that this limit is linear, whereas the blob test assumes that the limit is geometrically irregular; the latter is more robust if the species occupy geographical areas differing in extent, and the sample sizes differ (Glor & Warren, 2011). Identity tests assessed whether the models generated for the occurrence of two species differed statistically from those generated from random subsamples of their occurrences (Warren, Glor & Turelli, 2008). Background tests evaluated the environmental conditions that surrounded a species range, enabling assessment of whether the niches of two species were more similar to each other than to the available conditions (Warren, Glor & Turelli, 2008). We defined background regions as buffers of 500 km for the species and 250 km for the subspecies and natural micro-endemic *T. marginata*. These background regions were defined according to the distribution of each species or subspecies, maximizing the inclusion of environmental variation around the occurrence areas while minimizing the effect of non-informative regions (such as temperate-boreal biomes or deserts, not occupied by any species of the genus) (Acevedo et al., 2012).

The identity and background tests were conducted based on ENMs (Warren, Glor & Turelli, 2008) and an ordination method (ecospat) (Broennimann et al., 2012). The ecospat method has the advantage that it reduces the chances of model overfitting (Broennimann et al., 2012). We also estimated the Spearman rank correlation between environmental variation and predicted suitability (Warren et al., 2021). This correlation varies from −1 to 1, with 0 representing random correlation (Warren et al., 2021). These tests were implemented using generalized linear models and their statistical significance was assessed after 500 replicates (Warren et al., 2021). These analyses were conducted using the ENMTools package (Warren, Glor & Turelli, 2010) in R.

The evolutionary relationships among several species and subspecies of the genus *Testudo* were taken from a published molecular phylogeny (Graciá et al., 2017) (Fig. 2). The phylogenetic signal was determined only for a set of taxa in which the extent of divergence was quantified for the same genes (Fig. 2). The test of phylogenetic influence over environmental niche occupancy (defined by the same subset of low-correlated variables) was determined using Blomberg’s *K* (Blomberg, Garland Jr & Ives, 2003). Blomberg’s *K* values vary between 0 and ∞, where values of *K* < 1 represent less phylogenetic signal than that expected under Brownian motion (Blomberg, Garland Jr & Ives, 2003). Blomberg’s *K* provides an acceptable estimate of the phylogenetic signal even when sample sizes are limited, although is susceptible to false positives (Blomberg, Garland Jr & Ives, 2003; Münkemüller et al., 2012). The value of the phylogenetic metric was calculated after 10,000 resamplings of the climate matrix, thus avoiding the error associated with estimations based only on the mean value per species (Harmon & Losos, 2005). These calculations were performed using the phytools package (Revell, 2012) in R.

## Results

The scatter plot (Fig. 3) showed that the species occupy well-differentiated positions along the environmental gradient, from a mesophilic species (with high scores in the variables that describe precipitations), such as *T. hermanni*, other species that occupied an intermediate position (*T. graeca*, *T. marginata*), to xerophilic species such as *T. horsfieldii* and *T. kleinmanni*, that occupy one of the climate extremes (Fig. 3A). The subspecies show a tendency to overlap or occupy contiguous positions in the niche space (Fig. 3B), although the western group of *T. graeca* occupies a more limited niche than the eastern group of subspecies (Fig. 3B).

**Figure 4:**
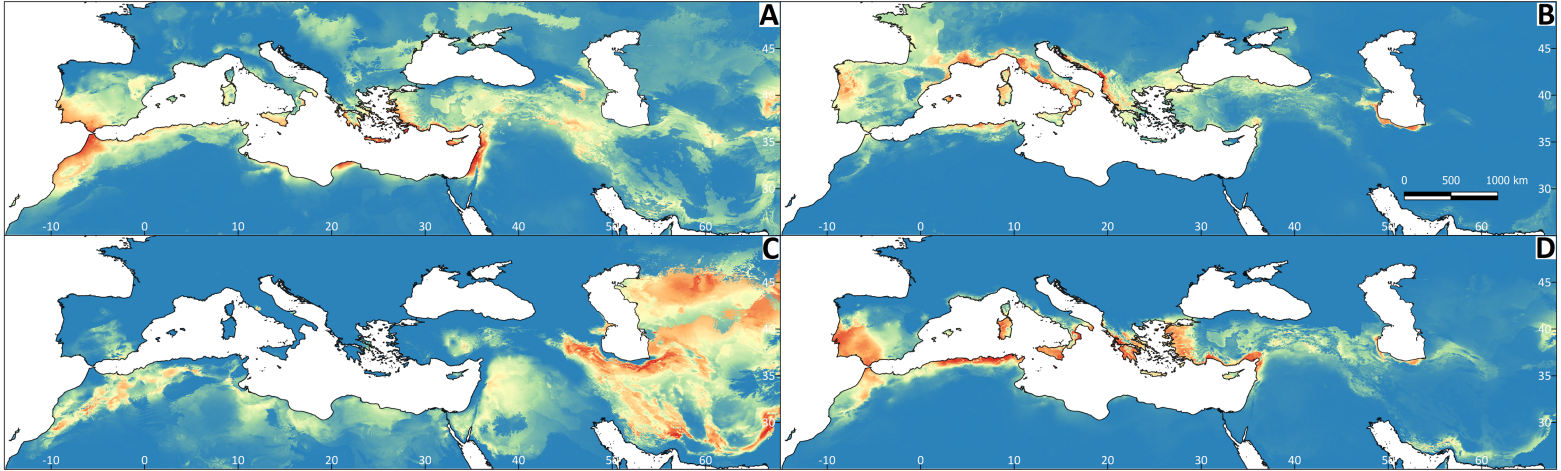
Results of the Maxent models representing the niche of *Testudo* species, obtained from climatic variables. The red colour indicates greater environmental suitability, and the blue colour reduced suitability. (A) *Testudo graeca*; (B) *Testudo hermanni*; (C) *Testudo horsfieldii*; (D) *Testudo marginata*.

**Figure 5:**
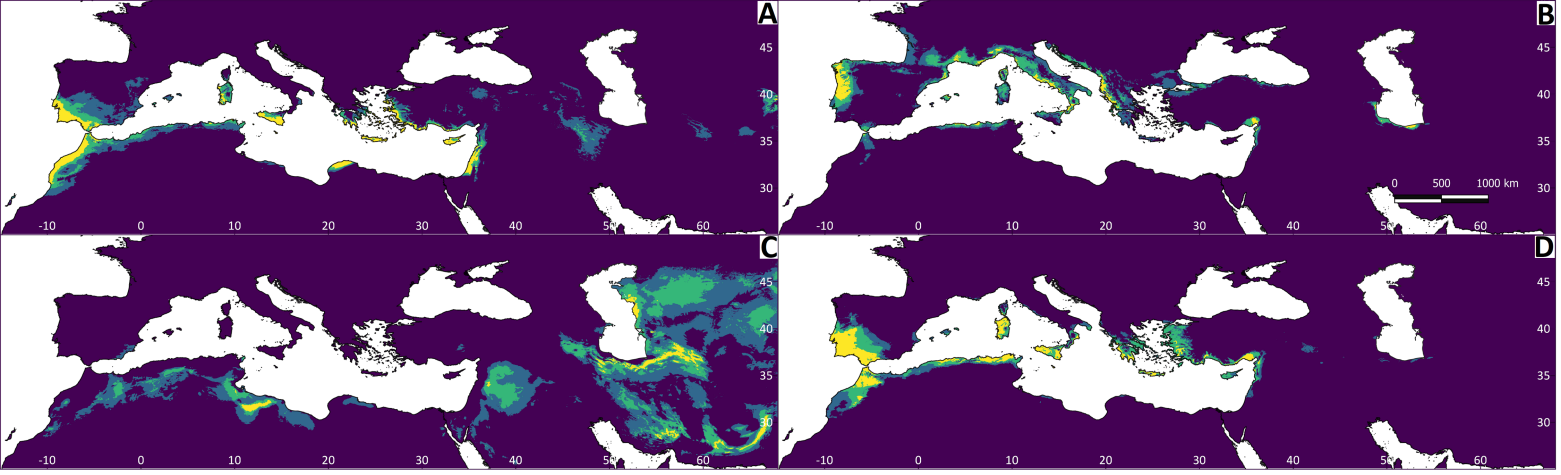
Regions most likely to represent stable climate refugia throughout the Pleistocene-Holocene periods (787 ka to 0 ya). The yellow colour indicates a higher probability (high suitability for at least five time periods), and the blue colour indicates high suitability for less than two time periods. (A) *Testudo graeca*; (B) *Testudo hermanni*; (C) *Testudo horsfieldii*; (D) *Testudo marginata*.

All Maxent models showed good performance (Appendix S2). The explanatory capacity of the climatic variables determining the species distributions differed significantly (Appendix S2). For *T. graeca* the variable having the greatest importance was annual precipitation (39.1%), for *T. hermanni* it was annual precipitation (77.9%), for *T. horsfieldii* it was the temperature of the wettest quarter and precipitation in the warmest quarter (29.1%), and for *T. marginata* it was precipitation in the warmest quarter (32.3%). The mapped results of the ENMs are shown in Fig. 4. The ENM projections identified several palaeoclimate refugia, which differed in most of the species (Fig. 5).

The results of the niche tests did not indicate that these species are niche conservative, both in the group of parapatric species and between sister species (*T. hermanni-T. horsfieldii*). The lack of statistical significance for most of the comparisons of the range breaking tests allows us to reject the hypothesis of sharp environmental variations at the species’ range boundaries (Table 1). In general, climatic niche overlap between species was lower than between subspecies, and species tend to occur in distinct climate niches (Tables 1 and 2). Similarly, species showed negative values or close to 0 in their correlations between climate variation and predicted suitability, while in some pairs of subspecies (*e.g.*, *T. g. graeca* and *T. g. marokkensis*) this correlation was positive, indicating similar environmental responses (Tables 1 and 2). The phylogenetic signal estimation indicated lability during the evolutionary processes for the genus *Testudo*, with absent or weak phylogenetic signal for the entire subset of bioclimatic variables included in the ENMs (*i.e.,* BIO8–BIO12, BIO15, and BIO18) (*K* ≤ 0.475; Table 3).

**Table 1:** Evaluation of ecological niche overlap between pairs of species of the genus *Testudo*.

		Overlap	Lin	Blob	Id	BG	Id_B_	BG_B_
*T. graeca* vs	D	0.656	0.368	0.368	0.370	0.343	0.386	0.327
*T. hermanni*	P		0.079	0.396	0.009	0.009	0.008	0.099
	Rho		−0.036	−0.036	−0.035	−0.051		
*T. graeca* vs	D	0.372	0.392	0.387	0.387	0.359	0.148	0.148
*T. horsfieldii*	P		0.416	0.396	0.009	0.009	0.001	0.174
	Rho		−0.430	−0.448	−0.448	−0.476		
*T. graeca* vs	D	0.226	0.251	0.251	0.245	0.243	0.361	0.361
*T. marginata*	P		0.475	0.347	0.009	0.009	0.596	0.073
	Rho		−0.034	−0.034	−0.052	−0.056		
*T. hermanni* vs	D	0.231	No	No	0.252	0.247	0.009	0.009
*T. horsfieldii*	P				0.004	0.004	0.001	0.430
	Rho				−0.277	−0.205		
*T. hermanni* vs	D	0.341	0.440	0.440	0.438	0.442	0.197	0.197
*T. marginata*	P		0.030	0.003	0.010	0.044	0.001	0.366
	Rho		0.548	0.548	0.547	0.692		
*T. horsfieldii* vs	D	0.130	No	No	0.241	0.235	0.145	0.155
*T. marginata*	P				0.005	0.015	0.019	0.145
	Rho				0.005	0.005		

**Notes.**

Lin linear range–breaking test Blob blob rangebreaking test Id identity test BG background test IdB identity test (ecospat) BGB background test (ecospat)

D Schoener’s D Rho Spearman rank correlation

No no geographic contact

**Table 2:** Evaluation of ecological niche overlap between pairs of species of the genus *Testudo*.

		Overlap	Lin	Blob	Id	BG	Id_B_	BG_B_
*T. g. graeca* vs	D	0.193	0.285	0.277	0.277	0.151	0.096	0.096
*T. g. marokkensis*	P		0.109	0.406	0.010	0.010	0.001	0.439
	Rho		0.338	0.330	0.330	0.129		
*T. g. marokkensis* vs	D	0.117	0.194	0.191	0.191	0.155	0.277	0.277
*T. g. whitei*	P		0.168	0.228	0.010	0.158	0.061	0.397
	Rho		−0.357	−0.354	−0.354	0.018		
*T. g. whitei* vs	D	0.623	0.313	0.316	0.321	0.298	0.452	0.452
*T. g. nabeulensis*	P		0.277	0.495	0.010	0.375	0.657	0.057
	Rho		0.286	0.273	0.284	0.375		
*T. graeca* western vs	D	0.309	No	No	0.158	0.146	0.230	0.230
*T. graeca* eastern	P				0.010	0.010	0.001	0.132
	Rho				−0.645	−0.668		
*T. g. ibera* vs	D	0.260	0.282	0.282	0.286	0.239	0.333	0.333
*T. g. terrestris*	P		0.149	0.069	0.010	0.040	0.838	0.021
	Rho		−0.089	−0.089	−0.081	−0.228		
*T. g. ibera* vs	D	0.250	0.260	0.263	0.264	0.336	0.116	0.116
*T. g. buxtoni*	P		0.416	0.227	0.010	0.099	0.085	0.350
	Rho		−0.618	−0.612	−0.614	−0.351		
*T. g. buxtoni* vs	D	0.174	0.221	0.218	0.219	0.235	0.057	0.057
*T. g. terrestris*	P		0.238	0.248	0.009	0.012	0.001	0.490
	Rho		−0.141	−0.141	−0.127	0.412		
*T. h. hermanni* vs	D	0.366	0.195	0.198	0.197	0.084	0.206	0.206
*T. h. boettgeri*	P		0.208	0.378	0.005	0.010	0.013	0.058
	Rho		−0.121	−0.133	−0.123	−0.504		

**Notes.**

Lin linear range-breaking test Blob blob rangebreaking test Id identity test BG background test IdB identity test (ecospat) BGB background test (ecospat)

D Schoener’s D Rho Spearman rank correlation

No no geographic contact

## Discussion

The combined results indicate niche partitioning among the species of the genus *Testudo*. The temperate and humid region is largely occupied by *T. hermanni* and as the conditions become more arid, the species progressively replace one another, appearing two species entirely adapted to extreme climates, cold steppes (*T. horsfieldii*) or specialized in sand semi-deserts (*T. kleinmanni*) (Bringsøe & Buskirk, 1998; Valakos et al., 2008; Bondarenko & Peregontsev, 2017; Arakelyan et al., 2018). Consistently, we found that climate niches are not phylogenetically conserved, as the values for Blomberg’s *K* in our analysis range from 0.263 to 0.475. Mapped projections of the ENMs showed marked differences in the climate niches of these species of tortoise, and niche overlap was generally low. The analyses also suggest that climate (and particularly annual precipitation, and precipitation and temperature of the warmest quarter) plays a key role in determining tortoise distribution in the study area, given the high predictive performance of the ENMs, as already indicated by previous studies involving the eastern clades of *T. graeca* (Turkozan, Karacaoğlu & Parham, 2021).

The analyses revealed that, in most cases, the geographical boundaries between these tortoise species and subspecies were not determined by rapid climate transitions. This implies that the species can partially differ in their niches, but also share zones of environmental tolerance, as shown by the ENM results. These regions (*e.g.*, the southern Balkans or Sardinia) are environmentally suitable for several species, and natural populations (or populations resulting from historical introductions; Vamberger et al., 2011) of two or three species coexist. In transitional regions, the species turnover may be determined by negative interspecific interactions or habitat properties, but they can also occur sympatrically (Wright, Steer & Hailey, 1988; Valakos et al., 2008).

The ENMs and ecospat identity tests did not show complete consistency. The results of the ecospat method were more conservative (*i.e.,* they rejected the null hypothesis of niche equivalency less frequently). We considered that the species are ecologically divergent if the results of the two identity tests were consistent. These tests showed a pattern of niche divergence for most of the species pairs, except for *T. graeca*–*T. marginata*. However, in this case, the environmental response was not convergent (*i.e.,* the values of the rank correlation coefficient were close to 0), and they were no more conservative than expected given their available conditions (*i.e.,* the background tests were not statistically significant).

**Table 3:** Estimation of the phylogenetic signal (Blomberg’s K) in the diversification of the ecological niche in the genus *Testudo*.

	Blomberg’s K
Temperature warmest quarter	0.365 ± 0.001
Temperature coldest quarter	0.475 ± 0.002
Temperature wettest quarter	0.352 ± 0.001
Temperature driest quarter	0.360 ± 0.002
Annual precipitation	0.437 ± 0.002
Precipitation seasonality	0.263 ± 0.001
Precipitation warmest quarter	0.467 ± 0.002

**Notes.**

The mean value and the standard error after 10,000 resamplings of the possible values of the niche for each species are shown.

The subspecies showed greater similarities in their climate niches, with pairs occurring in equivalent climate niches and with convergent environmental responses (for example, the *T. g. whitei*–*T. g. nabeulensis* pair). Lower niche divergence was expected between subspecies since their genetic separation spans smaller timescales (Peterson, 2011; Meynard et al., 2017).

Niche divergence was especially marked for the eastern and western groups of *T. graeca*, as indicated by significant differences in the identity tests and negative values for the rank correlation coefficient. Indeed, western populations of *T. graeca* are confined to warm and dry environments in southern Europe, whereas the species extends to colder regions in eastern Europe (Anadón et al., 2006; Buică, Iosif & Cogălniceanu, 2013). This high niche divergence observed between both clades would initially support their separation as species, as proposed by Van der Kuyl, Ballasina & Zorgdrager (2005). The North African subclade has evolved isolated in the southern Mediterranean, in a hot and arid region on the fringes of the Sahara, which could have reduced its cold tolerance, compared to Asian subspecies. However, it should be evaluated whether these differences in the realized niches between the western and eastern subclades of *T. graeca* also extend to the fundamental niches, through experimental mechanistic studies (Jiménez et al., 2019; Bujan et al., 2022).

Complementary to the niche tests, evaluation of the phylogenetic signal suggested a poorly conserved climate niche. This result contrasts with data for other reptile groups from the region, including lacertid lizards, which show a strong phylogenetic signal in species climate niches (Escoriza, Pascual & Mestre, 2021). However the power of Blomberg’s *K* tests is limited when using small phylogenies (Münkemüller et al., 2012), and future studies have to evaluate these evolutionary patterns with a larger number of tortoise species.

## Conclusions

The speciation process of tortoises from the genus *Testudo* is closely linked to regional climate history. The distribution of subspecies can be explained by the presence of stable climate refugia throughout the Pleistocene. Most species occupy well-defined and separate climate niches, reducing the chances of contact and interspecific interactions. In the case of occupation of equivalent niches, the species may occur in separate geographic regions (*e.g.*, the *T. graeca*–*T. marginata*).

##  Supplemental Information

10.7717/peerj.13702/supp-1 Appendix S1 Ocurrence sites coordinates (geodetic datum wgs 84) The thinned records represent the spatially independent coordinates +10 km.

10.7717/peerj.13702/supp-2 Appendix S2 Data sources and methodological results

10.7717/peerj.13702/supp-3 Appendix S3 ODMAP (Overview, Data, Model, Assessment and Prediction) protocol

## Additional Information and Declarations

### Data Availability

The following information was supplied regarding data availability: The raw measurements are available in the Supplementary File.
