# Supplementary material for: Niche diversification of Mediterranean and southwestern Asian tortoises
Source: PeerJ. 2022 Jul 11;10:e13702. doi: 10.7717/peerj.13702 (PMC9281595; doi:10.7717/peerj.13702)
Supplement: Appendix S2 [file peerj-10-13702-s002.docx]

Appendix 2. Bibliographic sources.

| Reference | Species |
| --- | --- |
| GBIF: https://www.gbif.org/ | All |
| iNaturalist: <https://www.inaturalist.org/Testudo> marginata | 1. *marginata* |
| Pierangelo Crucitti & Federica Emiliani (2012): Morphological variability of the Spurthighed Tortoise,Testudo graeca, in the Nemrut Volcano (Eastern Turkey), Zoology in the Middle East, 56:1, 19-26 | *T.graeca* |
| Diana Pop & Lucian Pârvulescu (2013): The northern most record of Hermann’s tortoise (Testudo hermanni boettgeri) in Romania, Annals of West University of Timisoara, ser. Biology, 15(1), 25-28 | *T. hermanni* |
| Ljiljana Tomovic, Rastko Ajtic & Ana Golubovic (2019): New records of Testudo graeca (Pallas, 1814) in Republic of Serbia, Bulletin of the Natural History Museum, 12, 209-215 | *T. graeca* |
| Markus Auer & Mario Herz (2021): Chelonians in Georgia, a travel report, with notes on the distribution of tortoises in the Caucasus Region, Radiata, 30:2, 4-21 | *T. graeca* |
| Durmus Cihan & Cemal Varol Tok (2014): Herpetofauna of the vicinity of Aksehir end Eber (Konya, Afyon), Turkey. Turkish Journal of Zoology, 38, 234-241 | *T. graeca* |
| Barbod Safei-Mahroo, Hanyeh Ghaffari, Hadi Fahimi, Siamak Broomand, Mahtab Yazdanian et al. (2015): The Herpetofauna of Iran: Checklist of Taxonomy, Distribution and Conservation Status. Asian Herpetological Research 6:4, 257-290 | *T. graeca* |
| Marcel Uhrin, Peter Havas, Martin Minarik, Karel Kodejs, Imrich Bugos, Stanislav Danko, Tomas Husak, Daniel Koleska & Daniel Jablonski (2016). Distribution updates to amphibian and reptile fauna for the Republic of Macedonia. Herpetology Notes 9, 201-220 | *T. graeca*  *T. hermanni* |
| Valentina Elena Moraru, Elena Buhaciuc, Dragos Stefan Mantoiu, Viorel Dumitru Gavril, Razvan Popescu-Mirceni & Alexandru Strugariu (2016): The spur-thighed tortoise (Testudo graeca ibera) in Romania: new locality records suggest more optimistic situation, North-Western Journal of Zoology 12:2, 396-398 | *T. graeca* |
| D.A. Bondarenko & E.A. Peregontsev (2017): Distribution of the central Asian tortoise Agrionemys horsfieldii (Gray, 1844) in Uzbekistan (Range, Regional and Lanrscape Distribution, Populations Density). Current Studies in Herpetology 17: 124-146. | *T. horsfieldii* |
| D.J. Harris, M.A. Carretero, J.C. Brito, A. Kaliontzopoulou, C. Pinho, A. Perera, R. Vasconcelos et al. (2008). Data on the distribution of the terrestrial herpetofauna of Moroco: records from 2001-2006. Herpetological Bulletin 103, 19-28 | *T. graeca* |
| Ana C. Andreu, Carmen Díaz-Paniagua & Claudia Keller (2000): La tortuga mora (Testudo graeca L.) en Doñana, Monografias de la asociación herpetológica española, vol. 5. | *T. graeca* |
| Henrik Bringsoe & James R. Buskirk (1998): Distribution of Testudo kleinmanni Lortet, 1883 and Testudo graeca Linnaeus, 1758 in the Negev Desert, southern Israel (Reptilia: Testudines: Testudinidae), Contributions to a Herpetologia Arabica 4(21), 23-30 | *T. kleinmanni*  *T. graeca* |
| Uwe Fritz, James Harris, Soumia Fahd, Rachid Rouag, Eva Graciá, Andrés Gimenez, Pavel Siroky, Mohsen Kalboussi, Tarek Jdeidi & Anna Hundsdörfer (2009): Mitochondrial phylogeography of Testudo graeca in the Western Mediterranean: Old complex divergence in North Africa and recent Arrival in Europe, Amphibia-Reptilia 30, 63-80 | *T. graeca* |
| Marine Arakelyan & James F. Parham (2008): The Geographic Distribution of Turtles in Armenia and the Nagorno-Karabakh Republic (Artsakh), Chelonian Conservation and Biology 7, 70-77 | *T. graeca* |
| Adel Ibrahim & Ivan Ineich (2005): Additional records to the herpetofauna of Nalut province, Libya, African Herp News 38, 2-8 | *T. graeca* |
| Yehudah L. Werner (2016): Reptile Life in the Land of Israel, Chimaira | *T. kleinmanni* |
| Marine Arakelyan, OguzTurkozan, NasimHezaveh, James F. Parham (2018). Ecomorphology of tortoises (Testudo graeca complex) from the Araks river valley. Russian Journal of Herpetology 25, 245-252 | *T. graeca* |
| Elias Tzoras, Alexis Panagiotopoulos, SeryiosPapaioannou (2021): First record of Testudo marginata (Chelonia: Testudinae) from the island of Samos, Greece (Eastern Greece), Parnassiana Archives, 9, 11-14 | *T. marginata*  *T. graeca* |
| Hossein Javanbakht, Flora Ihlow, Daniel Jablonski, Pavel Siroky, Uwe Fritz, Dennis Rödder, MozafarSharifi & Peter Mikulicek (2017). Genetic diversity and quaternary range dynamics in Iranian and Transcaucasian tortoises, Biological Journal of the Linnean Society, 121:3, 627-640 | *T. graeca* |
| Uwe Fritz, Anna K. Hundsdörfer, Pavel Siroky, Markus Auer, Hajigholi Kami, Jan Lehmann, Lyudmila F. Mazanaeva, OguzTürkozan & Michael Wink (2007). Phenotypic plasticity leads to incongruence between morphology-based taxonomy and genetic differentiation in western Palaearctic tortoises (Testudo graeca complex; Testudines, Testudinae). Amphibia-Reptilia 28, 97-121 | *T. graeca* |

Table 2. Best Maxent models selected by Akaike criterion (AICc), after evaluating several candidate models combining different features (L: linear; Q: Quadratic; H: Hinge; P: Product; T: Threshold) and regularization multipliers (RM). AUC, area under the curve. wAICc, Akaike weights. The best supported models are those with delta AICc equal to 0 and wAICc close to 1.

|  | Features | RM | AUC | AICc | Delta AICc | wAICc |
| --- | --- | --- | --- | --- | --- | --- |
| *T. graeca* | LQHPT | 2.5 | 0.925 | 5926.347 | 0.00 | 0.823 |
| *T. hermanni* | LQHPT | 2 | 0.975 | 2089.313 | 0.00 | 0.963 |
| *T. horsfieldii* | LQ  LQHP | 0.5  1.0 | 0.956  0.966 | 1628.965  1631.268 | 0.00  2.30 | 0.667  0.211 |
| *T. marginata* | LQ | 0.5 | 0.980 | 1156.233 | 0.00 | 0.999 |

Table 3. Permutation importance of environmental variables modelling the fundamental niche of *Testudo* species, using Maxent.

|  | *T. graeca* | *T. hermanni* | *T. horsfieldii* | *T. marginata* |
| --- | --- | --- | --- | --- |
| Temperature warmest quarter | 13.6 | 5.2 | 6.5 | 16.5 |
| Temperature coldest quarter | 1.3 | 1.5 | 2.7 | 10.1 |
| Temperature wettest quarter | 6.0 | 4.5 | **29.1** | 6.4 |
| Temperature driest quarter | 2.8 | 1.6 | 2.2 | 7.0 |
| Annual Precipitation | **39.8** | **77.9** | 25.0 | 17.7 |
| Precipitation seasonality | 14.1 | 1.5 | 5.4 | 10.0 |
| Precipitation warmest quarter | 22.3 | 7.7 | **29.1** | **32.3** |

**R. script for niche models**

**a) model evaluation**

library("ENMeval")

sitx<-read.table("MARGINATA.txt")

data(wrld_simpl)

plot(sitx, pch=21, cex=1, col = "red")

plot(wrld_simpl,add=T)

bio <- raster::getData("worldclim", var = "bio", res=5)#

bio <- bio[[c(10,11,8,9,12,13,18)]]

env_vars <- stack(bio)

e <- extent(-15, 80, 25, 50)

env_vars <- crop(env_vars, e)

dir.create('~/MaxEnt_Default')

xm <- maxent(env_vars, sitx, path='MaxEnt_Default')

r <- predict(xm, env_vars, progress='text')

plot(r)

def.results <- getFEATUs(paste('MaxEnt_Default',"/maxent.html",sep=''))

def.results <- strsplit(def.results, " ")[[1]]

def.results <- lapply(def.results, function(x) gsub("hinge", "H", x))

def.results <- lapply(def.results, function(x) gsub("linear", "L", x))

def.results <- lapply(def.results, function(x) gsub("product", "P", x))

def.results <- lapply(def.results, function(x) gsub("threshold", "T", x))

def.results <- lapply(def.results, function(x) gsub("quadratic", "Q", x))

def.results <- lapply(def.results, function(x) paste(x, collapse = ""))

def.results <- paste(unlist(def.results),collapse='')

def.results

dir.create(ENMeval')

eval.results <- ENMevaluate(sitx=sitx, env=env_vars, RMvalues=seq(0.5, 4, 0.5),

featu=c("L", "LQ", "H", "LQH", "LQHP", "LQHPT"), method='block', algorithm='maxent.jar')

aicmods <- which(eval.results@results$AICc == min(na.omit(eval.results@results$AICc)))

eval.results@results[aicmods,]

aicmods <- which(eval.results@results$AICc == min(na.omit(eval.results@results$AICc)))[1]

aicmods <- eval.results@results[aicmods,]

FEATU_best <- as.character(aicmods$features[1])

rm_best <- aicmods$rm

maxent.args <- make.args(RMvalues = rm_best, featu = FEATU_best)

mx_best <- maxent(env_vars, sitx, args=maxent.args[[1]],

overight=T)

r_best <- predict(mx_best, env_vars, overwrite=TRUE, progress = 'text')

**b) niche tests**

library(ENMTools)

env <- raster::getData('worldclim', var='bio', res= 5)

env <- crop(env, extent(-15, 80, 25, 50))

graeca <- enmtools.species()

env <- env[[c("bio8", "bio9", "bio10", "bio11", "bio12", "bio15","bio18")]]

id.glm <- identity.test(species.1 = graeca, env = env, type = "glm", nreps = 500)
