# Supplementary material for: Niche diversification of Mediterranean and southwestern Asian tortoises
Source: PeerJ. 2022 Jul 11;10:e13702. doi: 10.7717/peerj.13702 (PMC9281595; doi:10.7717/peerj.13702)
Supplement: Appendix S3 [file peerj-10-13702-s003.docx]

Niche diversification of Mediterranean and South-Western Asian tortoises

– ODMAP Protocol –

Daniel Escoriza

2022-06-09

## Overview

#### Authorship

Contact : [daniel_escoriza@hotmail.com](mailto:daniel_escoriza@hotmail.com)

Study link: <https://peerj.com/>

#### Model objective

Model objective: Inference and explanation

#### Focal Taxon

Focal Taxon: Mediterranean Tortoises

#### Location

Location: Mediterranean basin; south-western Asia

#### Scale of Analysis

Spatial extent: -15, 80, 25, 50 (xmin, xmax, ymin, ymax)

Spatial resolution: 1

Temporal extent: Pliocene to present

Temporal resolution: years

Boundary: rectangle

#### Biodiversity data

Observation type: field survey, citizen science

Response data type: point occurrence

#### Predictors

Predictor types: climatic

#### Hypotheses

Hypotheses: climate partitioning among species

#### Assumptions

Model assumptions: Species–environment equilibrium (climate determines species fitness)

#### Algorithms

Modelling techniques: maxent, glm

Model complexity: assessment of the best candidate model features to fit the dataset

Model averaging: ensemble modelling

#### Workflow

Model workflow: remove multicollinear variables-remove spatial redundancy-select best maxent model

#### Software

Software: R, enmval, Maxent

Code availability: scripts available as supplementary material

Data availability: data available as supplementary material and data links to the sources

## Data

#### Biodiversity data

Taxon names: Testudo graeca, Testudo hermanni, Testudo horsfieldii, Testudo kleinmanni, Testudo marginata

Taxonomic reference system: Linnean-Rhodin AGJ, Iverson JB, Bour R, Fritz U, Georges A, Shaffer HB, van Dijk PP. 2021. Turtles of the World: Annotated Checklist and Atlas of Taxonomy, Synonymy, Distribution, and Conservation Status (9th Ed.). Chelonian Research Monographs 8:1-472. DOI: 10.3854/crm.8.checklist.atlas.v9.2021.

Ecological level: species

Data sources: <https://www.gbif.org/> <https://www.inaturalist.org/>

Sampling design: uniform

Sample size: T. graeca (274 records), T. hermanni (103), T. horsfieldii (77), T. kleinmanni (4), and T. marginata (61)

Clipping: western Palaearctic

Scaling: spatial thinning 10 km

Cleaning: We selected from the GBIF those records added since the year 2000, with fine spatial resolution (error < 1000 m), far from cities and within the known ranges of the species, as are defined by Bonin, Devaux & Dupré (2006).

Absence data: not applicable

Background data: We defined background regions as buffers of 500 km for the species and 250 km for the subspecies and natural micro-endemic T. marginata. These background regions were defined according to the distribution of each species or subspecies, maximizing the inclusion of environmental variation around the occurrence areas while minimizing the effect of non-informative regions (such as temperate-boreal biomes or deserts, not occupied by any species of the genus) .

Errors and biases: Data from open databases is subject to these errors: misidentification potential, geo-referencing errors, sampling bias

#### Predictor variables

Predictor variables: Temperature warmest quarter Temperature coldest quarter Temperature wettest quarter Temperature driest quarter Annual Precipitation Precipitation seasonality Precipitation warmest quarter

Data sources: Worlclim 2 (June 2021): <http://www.worldclim.com/version2> <http://www.worldclim.com/paleo-climate1> (June 2021)

Spatial extent: -15, 80, 25, 50 (xmin, xmax, ymin, ymax)

Spatial resolution: 1 km

Coordinate reference system: wgs 84

Temporal extent: 1970-2000

Temporal resolution: not applicable

Data processing: not applicable

Errors and biases: not applicable

Dimension reduction: Model complexity was reduced by estimating the increase in the variance inflation factor (VIF)

#### Transfer data

Spatial extent: -15, 80, 25, 50 (xmin, xmax, ymin, ymax)

Spatial resolution: 1 km

Temporal extent: Pliocene-Holocene

Temporal resolution: not applicable

## Model

#### Variable pre-selection

Variable pre-selection: We started with a simple model including only bio 10 (mean temperature of warmest quarter), bio 11 (mean temperature of coldest quarter), and bio 12 (annual precipitation). These variables were initially chosen because of their relevance in the life cycles of tortoises, and they included summer and winter temperatures (specifically associated with embryonic development and hibernation) and moisture availability.

#### Multicollinearity

Multicollinearity: Model complexity was reduced by estimating the increase in the variance inflation factor (VIF).

#### Model settings

maxent: featureSet (LQHPT LQHPT LQ LQHP LQ), featureRule (default), regularizationMultiplierSet (2.5 2 0.5 1.0 0.5), regularizationRule (default), convergenceThresholdSet (default), samplingBiasRule (default), samplingBiasNotes (default), targetGroupSampleSize (default), offsetSet (default), offsetRule (default), expertMapProbSet (default), expertMapProbRule (default), expertMapRateSet (default), expertMapRateRule (default), expertMapSkewSet (default), expertMapSkewRule (default), expertMapShiftSet (default), expertMapShiftRule (default)

glm: family (Logistic), formula (pres ~ bio8+bio9+bio10+bio11+bio12+bio15+bio18)

#### Model estimates

Coefficients: not applicable

Parameter uncertainty: resampling

Variable importance: resampling

#### Model selection - model averaging - ensembles

Model selection: information-theoretic approach for variable selection

Model averaging: not applicable

Model ensembles: ENMs: The features and the RM for the optimal model were used to generate 30 replicates in Maxent 3.4.4 (Philips, Anderson & Schapire, 2006), calibrated using 75% of the species occurrences. GLMs: number of replicates = 9999, number of background points for models = 10000

#### Analysis and Correction of non-independence

Spatial autocorrelation: spatial thinning of occurrences

Temporal autocorrelation: not applicable

Nested data: not applicable

## Assessment

#### Performance statistics

Performance on training data: AIC, AUC

#### Plausibility check

Response shapes: partial response plots
